# Pediatric Anti-*N*-Methyl-d-Aspartate Receptor Encephalitis: A Review with Pooled Analysis and Critical Care Emphasis

**DOI:** 10.3389/fped.2017.00250

**Published:** 2017-11-24

**Authors:** Kenneth E. Remy, Jason W. Custer, Joshua Cappell, Cortney B. Foster, Nan A. Garber, L. Kyle Walker, Liliana Simon, Dayanand Bagdure

**Affiliations:** ^1^Division of Pediatric Critical Care Medicine, Department of Pediatrics, University of Maryland School of Medicine, Baltimore, MD, United States; ^2^Critical Care Medicine Department, Clinical Center, The National Institutes of Health, Bethesda, MD, United States; ^3^Division of Pediatric Critical Care, Department of Pediatrics, Washington University School of Medicine, St. Louis, MO, United States; ^4^Divisions of Pediatric Neurology and Critical Care, Departments of Neurology and Pediatrics, Morgan Stanley Children’s Hospital, Columbia University College of Physicians and Surgeons, New York, NY, United States

**Keywords:** *N*-methyl-d-aspartate receptor, *N*-methyl-d-aspartate, encephalitis, autoimmune, critical care, pediatrics, paraneoplastic

## Abstract

**Purpose:**

Anti-*N*-methyl-d-aspartate receptor (NMDAR) encephalitis is being recognized with increasing frequency among children. Given the paucity of evidence to guide the critical care management of these complex patients, we provide a comprehensive review of the literature with pooled analysis of published case reports and case series.

**Methods:**

We performed a comprehensive literature search using PubMed, Scopus, EMBASE, and Web of Science for relevant published studies. The literature search was conducted using the terms NMDA, anti-NMDA, Anti-*N*-methyl-d-aspartate, pediatric encephalitis, and anti-NMDAR and included articles published between 2005 and May 1, 2016.

**Results:**

Forty-eight references met inclusion criteria accounting for 373 cases. For first-line treatments, 335 (89.8%) received high-dose corticosteroids, 296 received intravenous immunoglobulin (79.3%), and 116 (31%) received therapeutic plasma exchange. In these, 187 children (50.1%) had a full recovery with only minor deficits, 174 patients (46.7%) had partial recovery with major deficits, and 12 children died. In addition, 14 patients were reported to require mechanical ventilation.

**Conclusion:**

Anti-NMDA encephalitis is a formidable disease with great variation in clinical presentation and response to treatment. With early recognition of this second most common cause of pediatric encephalitis, a multidisciplinary approach by physicians may provide earlier access to first- and second-line therapies. Future studies are needed to examine the efficacy of these current therapeutic strategies on long-term morbidity.

## Introduction

Pediatric critical care physicians are increasingly called upon to diagnose, manage, and treat children with anti-*N*-methyl-d-aspartate receptor (anti-NMDAR) encephalitis. Anti-NMDAR encephalitis was originally described in 2005 in four women with ovarian teratomas, presenting with seizures, acute psychiatric disturbances, cognitive deficits, decreased sensorium, autonomic instability, and hypoventilation (1). Dalmau et al. in 2007 diagnosed these women and eight others after demonstrating specific autoantibodies to the NMDAR; namely, a neuronal cell surface protein that was shown to be the NR1 subunit of the receptor (2).

Since the first reported cases and discovery of anti-NMDAR encephalitis, this disease has surpassed all single viral etiologies and has been recognized as the second most common entity after the mixed disturbance encephalomyelitis, acute demyelinating encephalitis (2, 3). The estimated mortality for anti-NMDAR encephalitis in one series of 100 adults with a median age of 23 years was 4% (4). Although over 600 adult and pediatric cases have been reported in the literature with 2 case series of 15 patients and 77 patients among critically ill adults, further critical care-focused considerations have been lacking (5–12). Nonetheless, some authors have suggested that earlier treatment in children may result in better outcomes (13–15). We reviewed the literature to evaluate the use of corticosteroids and intravenous immunoglobulin (IVIG) as a first-line therapy for patients with anti-NMDAR encephalitis. Next, we provide a pooled analysis of reviewed data and critical care considerations based on literature review for critically ill children with anti-NMDAR encephalitis.

## Materials and Methods

We conducted a search of PubMed, Scopus, EMBASE, and Web of Science to identify relevant published studies and abstracts and then conducted a systematic, narrative review of potentially relevant sources. The literature search was conducted using the terms NMDA, anti-NMDA, anti-NMDAR, pediatric encephalitis, and anti-NMDAR and included articles published between January 1, 2005, and May 1, 2016. Furthermore, the search was limited to “humans,” children, and abstracts or reports published in English, and screening was performed by the first author (Kenneth E. Remy). Our search criteria are included in the supplemental information. Inclusion criteria were any clinical study or report in which subjects (1) demonstrated anti-NMDA receptor encephalitis by positive serum and/or cerebrospinal fluid (CSF) anti-NMDAR and (2) were younger than 19 years (intended age, 0–18 years). Exclusion criteria included (1) the absence of a laboratory data to support the diagnosis, i.e., no evidence of serum and/or CSF NMDAR antibodies; (2) adults older than 18 years; or (3) publications not in English. Since the majority of this information was individual case reports, this information was combined and tabulated to document current practice. The variables collected included age; sex; presence of positive CSF or blood anti-NMDA receptor titers; use of first-line therapies including high-dose corticosteroids, IVIG, or plasma exchange; use of second-line therapies such as rituximab or cyclophosphamide; and need for mechanical ventilation. Outcome measures were modified from previous studies and classified as complete recovery with only mild deficit, partial recovery with significant deficit, and death (8, 16). One of the authors (Kenneth E. Remy) retrospectively assigned all patients to one of these outcome groups. As this review was performed on published reports and lacked patient identifying data, the University of Maryland Institutional Review Board waived the need for approval.

## Results

**Figure 1:**
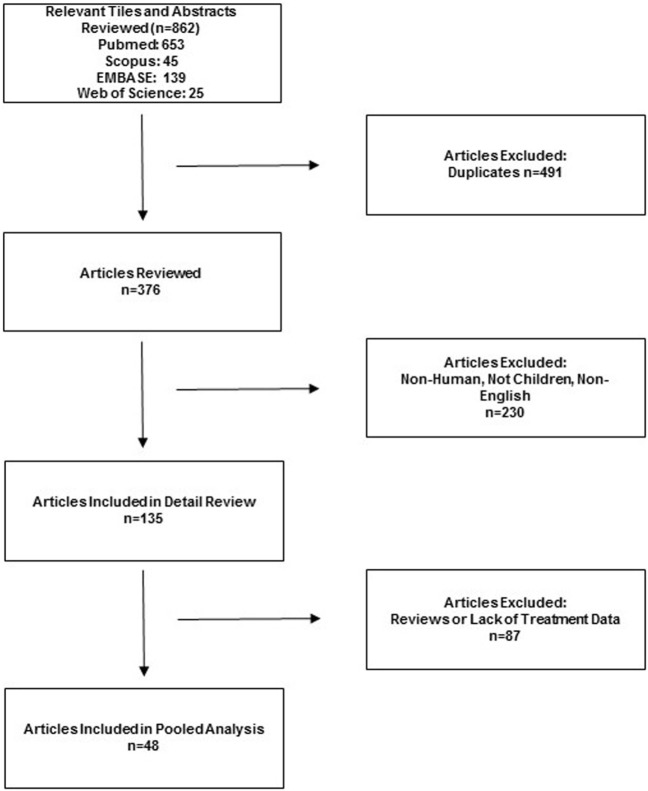
Flow diagram of the published articles evaluated for inclusion in pooled analysis.

The search identified 862 potentially relevant studies, and 201 were selected for data extraction. From these, 48 references met inclusion criteria, including 27 individual case reports and 21 case series (Figure 1) (4, 10–12, 14, 15, 17–57). Two prospective cohort studies were found (45, 53). One large retrospective cohort series accounted for 211 patients (14). The presence of both clinical diversity and methodological diversity precluded combining the study reports in a meta-analysis. Whenever possible, authors were electronically mailed for missing data.

A total of 382 patients were included; however, incomplete data were included in the case reports for 9 patients. Thus, 373 patients with a mean age of 9.98 years (range, 3 months–18 years) were included in our pooled data analysis presented in Table 1. Larger case series that included only means and range of ages precluded calculation of SD of mean age. The pooled analysis included 254 females (68.1%) and 119 (31.9%) males (9 reports did not include sex). Subjects represented nine different countries. Sixty-five of 66 patients with evidence of teratoma (17.6%) were females, as described in the original case reports (1). Anti-NMDA titers were positive in the CSF and serum of 327 (87.6%) and 276 (74%) patients, respectively.

**Table 1:** Pooled analysis.

Total patients	373 children
Sex	254F (68.1%) 119 M (31.9%)
Mean age	9.98 years (range, 3–18 years)
Teratoma or malignancy present	66 (17.6%)
CSF positive for anti-NMDAR	327 (87.6%)
Serum positive for anti-NMDAR	276 (74%)
**First-line treatments**
High-dose corticosteroids	335 (89.8%)
Intravenous immunoglobulin	296 (79.3%)
Plasma exchange	116 (31%)
**Second-line treatments**
Rituximab	87 (23.3%)
Cyclophosphamide	62 (16.6%)
**Third-line treatments**
Electroconvulsive therapy	5 (1.3%)
Mechanical ventilation	14 (3.8%)
**Outcomes**
Complete recovery with minor deficits	187 (50.1%)
Partial recovery with major deficits	174 (46.7%)
Death	12 (3.2%)

For first-line treatments (as reported as first agent given or delineated in report), 335 (89.8%) received high-dose corticosteroids, 296 received IVIG (79.3%), and 116 (31%) received therapeutic plasma exchange. Five patients received electroconvulsive therapy (ECT) (34, 46, 50–52), and 14 children required mechanical ventilation (11, 12, 22, 28, 37, 38, 42, 44, 52, 53, 55, 56). There were five reports that explicitly defined the use of intensive care or pediatric intensive care unit although some reports alluded to need for “advanced” therapies (30, 34, 38, 54, 55). In reported outcomes, 187 children (50.1%) had a full recovery with only minor deficits, while 174 patients (46.7%) had partial recovery with major deficits at 1-year follow-up. Twelve children (3.2%) died subsequent to their disease.

To best define the use of first- and second-line therapies, we next examined the reports that provided delineation between initiations of specific therapies. In 15 reports of 20 patients that defined a time period before first agent and initiation of second agent, 13 patients received high corticosteroids administered on mean day 8.71 (SD ± 3.2) and 7 patients received IVIG on mean day 7.57 (SD ± 7.7) (19, 21, 25, 27–30, 32, 36, 37, 41, 42, 49, 50, 58). The mean time course from reported first agent to second in these 15 reports was 11.4 days (SD ± 15.6). Second-line therapies included rituximab in 87 patients (23.3%) and cyclophosphamide 62 patients (16.6%). In this cohort of 15 reports (*n* = 19 patients), clinical duration of symptoms began 31.3 ± 20.1 days prior to admission (range, 1–90 days). Time to complete resolution of symptoms was 115.6 ± 86 days (range, 21–279 days; *n* = 15 patients), and four patients did not have complete resolution.

## Discussion

We pooled data from all relevant case reports, case series, and available retrospective cohort studies to better characterize the disease epidemiology, treatment modalities, and clinical outcomes of children with anti-NMDAR encephalitis.

### Epidemiology

Anti-*N*-methyl-d-aspartate receptor encephalitis affects both males and females, with a higher incidence among females (75% cases) (8, 32, 52, 59–62). Although it was originally described as a paraneoplastic disease with 58% patients showing evidence of an underlying tumor (most commonly ovarian teratoma), published literature reports only 31 and 9% of children younger than 18 years and younger than 14 years, respectively, had evidence of tumor (8, 16). As non-Caucasians have a higher incidence of ovarian teratomas, they also have higher incidence of anti-NMDAR encephalitis (32, 59). This association has been described in children as young as 3 months of age; however, most of the reported cases are adolescents (21, 61, 63, 64). In our pooled analysis, we have identified similar findings to those reported previously, with almost 70% of cases being female, an average age of 10 years, and 18% patients having a teratoma.

Despite these data, the diagnosis of anti-NMDAR encephalitis is still uncommon with many cases designated as idiopathic and possible or probable. Only 79 patients had identifiable diagnoses in the California Encephalitis Project of 761 cases, with 32 children having anti-NMDAR encephalitis (4.2%) and 30 children with enteroviral encephalitis (3.9%). Within this cohort, DuBray et al. described that 11% of children with encephalitis at her single center (compared to the entire centers in the project) occurred secondary to *Mycoplasma* infection, with a slightly lower incidence of 3.6% resulting from anti-NMDAR antibodies (65). Of interest, one-half of the cases with idiopathic psychiatric symptoms had antibodies against NMDAR (3, 22). This raises the question whether children with postinfectious encephalitis (i.e., HSV and *Mycoplasma*) and without tumor develop anti-NMDAR antibodies as parts of a larger, nonspecific immune response to these pathogens. The inciting infection may trigger antibodies directed against NMDAR, and thus, unusual presentations of viral encephalitis should be screened for concomitant autoimmune encephalitis (17, 42). Awareness of this disease and the availability of diagnostic molecular tests may improve our ability to diagnose the underlying etiologies of pediatric encephalitides.

### Clinical Course

Some authors have described anti-NMDAR encephalitis as having three clinical stages; a prodromal stage, an early (psychotic and/or seizure phase), and a late (hyperkinetic) phase (62, 66). Most interestingly, serum and CSF antibody titers seem to correlate with the disease course: a decline in titers coincides with improvement in symptoms (16). In the prodromal stage, symptoms include fever, nausea, vomiting, or upper respiratory tract infection-like symptoms (4, 22, 31, 67). This prodrome may last up to 2 weeks in 70% of patients (2, 31). During the early stage, patients may develop psychiatric symptoms such as fear, delusions, mania, and/or paranoia—manifesting in children as behavioral disturbances and tantrums rather than an underlying pathologic process. Children are more likely than adults to present with seizures, dystonia, or status epilepticus (11). Within 3–4 weeks, a more fulminant stage develops. Rapid disintegration of speech and language, hyperactivity, mutism, and irritability are often seen. Progression to decreased responsiveness, catatonia, autonomic instability, cardiac arrhythmias, hypoventilation, and uncoordinated respiration can lead to rapid deterioration and need for *critical care* interventions. Autonomic dysregulation and hypoventilation are less common in children than adults, while speech dysfunction appears more frequently (22, 68). During treatment, recovery has been described as reversal of symptoms in the reverse order of presentation (16).

### Diagnostic Evaluation

Once clinical suspicion is raised, all patients presenting with encephalitis should be evaluated for anti-NMDAR antibodies and receive imaging studies to exclude the presence of a teratoma (62, 69). Definitive diagnosis is made by CSF evidence of anti-NMDAR antibodies. CSF studies usually demonstrate an elevated white blood cell count, lymphocytic pleocytosis in 87% of children, and oligoclonal bands in 60% of patients (16, 31, 69). In our analysis, most patients had positive serum and/or CSF antibodies to NMDAR. However, one must interpret our findings with caution as reporting bias may falsely elevate positive results. While examining for anti-NMDAR encephalitis, one should also consider other encephalitides including AMPA-R, metabotropic glutamate receptor, voltage-gated potassium channels (LGI1, CASPR2, or contactin-2), anti-Hu, anti-CV2, anti-CRM, anti-P5, anti-MA proteins, voltage-gated calcium channel defects, GABA^B^-R, glycine-R, and GABA^A^-R defects (53, 70, 71).

### Clinical Management

When there is high clinical suspicion for the disease, rapid treatment should occur in a stepwise fashion as confirmatory testing is not performed at some centers and may take days to weeks (62, 66). The known pathophysiology and current (low-grade) evidence suggests that starting therapy earlier may lead to better outcomes. With appropriate treatment, substantial improvement or complete recovery can occur in up to 75% of patients (4, 14). However, relapses may occur in approximately 25% of children even after resolution (8). Surgical removal of a teratoma or other tumor is necessary but should not delay initiation of definitive therapy. In our small cohort of reports that defined clinical symptom time course of presentation and resolution after treatment, there was great variation among different aged patients and response to therapies. This makes generalizations of presentation and treatment findings difficult to assess. However, from current evidence, first-line therapies have included plasma exchange, IVIG, or high-dose corticosteroids. Based on all available information, it seems that IVIG therapy with 0.4 g/kg daily for 5 days or 1 g/kg on day 1 followed by 0.5 g/kg/day for 2 additional days and methylprednisolone 1 g (adults) or 30 mg/kg (children up to 40 kg) daily for 3 days or daily plasma exchange for six cycles would likely have favorable clinical effects (Table 2). In refractory cases or critically ill patients, second-line immunomodulation with rituximab or cyclophosphamide may be attempted (2, 4, 8, 27, 31). In adults and children, combined second-line therapies of rituximab 375 mg/m^2^ weekly for 4 weeks and cyclophosphamide 750 mg/m^2^ monthly have been proposed (27, 51). After therapy is completed, some authors recommend yearly screening with pelvic magnetic resonance imaging to assess for ovarian tumors (8, 32, 69). Details of critical care management for individual patients were unavailable for us to define treatment benefit with individual treatment approaches. Future prospective studies should evaluate the need and indications for ICU admission, duration of ICU care, complications associated with the disease and/or treatments, and evaluation of all-cause mortality among patients with this disease.

**Table 2:** Proposed therapies for anti-NMDAR encephalitis.

First-line therapies (if tumor is present, begin after removal)	Second-line therapies (if disease is refractory after 10 days)
IVIG 0.4 g/kg for 5 days and methylprednisolone 30 mg/kg daily for 5 days or plasma exchange daily for six cycles	Rituximab 375 mg/m^2^ weekly for 4 weeks and cyclophosphamide 750 mg/m^2^ monthly (duration is based on clinical improvement)

### Critical Care Considerations

Children with significant neurological symptoms should be initially managed in an ICU as autonomic dysfunction, airway protection, hypoventilation, cardiac arrhythmia, or hyperkinetic crisis may occur. Pediatric critical care requires an interdisciplinary team including neurologists, psychiatrists, cardiologists, nutritionists, physical therapists, social workers, physiatrists, pastoral care, and child life experts. Symptom-guided therapies are listed in Table 3.

**Table 3:** Intensive care symptom-guided therapies.

Symptoms	First-line therapy	Second-line therapy	Refractory therapy
Agitation	Trihexyphenidyl (0.02–0.06 mg/kg or 1 mg 1–2 times daily) and low-dose opioids (fentanyl 1 μg/kg/h infusion or bolus)	Dexmedetomidine infusion (0.2 μg–1.5 μg/kg/min)	Propofol infusion (10–30 μg/kg/min)

Catatonia autonomic dysfunction bradycardia	Glycopyrrolate (0.004–0.01 mg/kg q4–8 h prn: maximum, 0.1–0.2 mg/dose, 0.8 mg/day)	Theophylline (begin oral 300 mg/day divided q4–6 h up to a maximum dose 600 mg/24 h)	Electroconvulsive therapy

Hypoventilation			Tracheostomy (if ventilated for >4 weeks)

Additional considerations			Gastric-tube placement
			Early physical therapy

There are two main variations of instability that require critical care management: a neurologic type with catatonic or hyperkinetic crises and one that manifests with autonomic and hemodynamic instability (10, 27, 50). Although 84% of children have hyperkinetic movements, the literature is lacking in effective therapies (8). Valproic acid, gabapentin, and lithium have been used for mood dysregulation (25, 28, 31, 72). Clonidine, trazodone, and benzodiazepines target sleep cycle dysregulation, and phenobarbital, trihexyphenidyl (Artane™), and opioids are used for extreme agitation (10, 25, 28, 30, 38, 50). For catatonia, benzodiazepines or rarely ECTs have been reported (8, 16, 51, 73, 74). Routine management of agitation may include low-dose opioids, other sedatives, anxiolytics, or anti-psychotic drugs. For refractory agitation, methotrimeprazine or dexmedetomidine infusion may be beneficial for both intubated and non-intubated patients. For continued refractory symptoms, mechanical ventilation and low-dose propofol or ketamine infusions may be beneficial, although used with extreme caution. Careful attention and laboratory surveillance are needed to avoid propofol infusion syndrome, especially for children younger than 5 years and those receiving propofol for longer than 24 hours (75–78). Barbiturates and dexmedetomidine should be used at the lowest possible dosing to avoid bradycardia. Non-pharmacological therapies (noise reduction, adjusting room ambiance) should be employed to avoid agitation. Finally, muscle relaxants should be avoided to avoid ICU myopathies, especially with concomitant corticosteroid therapy (79).

The autonomic and hemodynamic instability can be difficult to manage. Resting tachycardia is widely reported in multiple case studies of pediatric patients (4, 8, 11). However, severe bradycardia and asystole events may be exacerbated by vagal stimulation including endotracheal tube suctioning or bowel movements (36, 80). Foley catheter use, bowel regimens to prevent constipation, and careful suctioning of the endotracheal tube are essential to minimize parasympathetic stimulation. Bradycardia events were also associated with seizures in this patient population and continuous electroencephalogram should be considered to capture these events (81).

Chronic use of medications such as glycopyrrolate or theophylline may be considered for preventing severe bradycardia; these medications have been successful in other disease processes to prevent symptomatic bradycardia (82). If severe bradycardia is frequent and unresponsive to medical therapies, an implantable pacemaker may be considered (83). Brain imaging to diagnose hydrocephalus may be indicated with new onset of bradycardia events in some patients (69).

Autonomic dysfunction also manifests as impaired temperature or blood pressure regulation. While patients are maintained on immunosuppressant therapies, infectious etiologies for hypothermia/hyperthermia must be considered. We have observed that movement disorders are often exacerbated during the febrile state; aggressive temperature control should be implemented with antipyretics, environmental cooling, and cooling blankets as indicated.

Hypoventilation in ICU patients with anti-NMDAR encephalitis may make it difficult to wean from mechanical ventilation. Ventilator weaning in these patients is further complicated by delirium, agitation, and movement disorders that may require heavy sedation. Placement of a tracheostomy and gastrostomy tube in the third week after diagnosis may improve patient safety and allow for less sedation and earlier rehabilitation.

Once medically feasible, it is paramount to begin a comprehensive rehabilitation program including physical, occupational, speech, and dysphagia therapy to improve long-term outcomes (24, 84). Functional decline in motor skills and cognition is common among patients with anti-NMDAR encephalitis and physical therapists with physiatrists should be engaged early in their management.

The available heterogeneous data impose methodological limitations for pooled analysis. Thus, one weakness of this review is the lack of statistical analysis for successful treatment or prognostic indicators. Our recommendations are limited to available low-grade evidence. However, this study provides a first step toward conducting randomized controlled trials to evaluate the efficacy of potential therapies.

## Conclusion

Anti-*N*-methyl-d-aspartate receptor encephalitis is a commonly encountered adult and pediatric critical care diagnosis. Careful understanding of the clinical features of the disease, diagnosis, and treatment options may improve the care of these complex patients. Early rehabilitation and careful consideration for tracheostomy and gastrostomy placement may further improve functional outcomes. Future studies investigating both pharmacologic approaches and non-pharmacologic interventions of autonomic dysfunction, movement disorders, and cardiac arrhythmias will be important in improving patient care and outcomes.

## Author Contributions

KR, JWC, JC, CF, NG, LW, LS, and DB all had direct involvement in the design, writing, and editing of this manuscript. KR performed the pooled analysis.

## Conflict of Interest Statement

The authors declare that the research was conducted in the absence of any commercial or financial relationships that could be construed as a potential conflict of interest.

## Abbreviations

CT, Cat scan; CNS, central nervous system; CPR, cardiopulmonary resuscitation; CSF, cerebrospinal fluid; EEG, electroencephalogram; IVIG, intravenous immunoglobulin; MRI, magnetic resonance imaging; NMDAR, *N*-methyl-d-aspartate receptor; PICU, pediatric intensive care unit.
